# Langmuir Probe Measurements in the Gaseous Electronics Conference RF Reference Cell

**DOI:** 10.6028/jres.100.031

**Published:** 1995

**Authors:** M. B. Hopkins

**Affiliations:** Physics Department, Dublin City University, Dublin 9, Ireland

**Keywords:** GEC Cell, Langmuir probe, plasma diagnostic

## Abstract

The use of a Langmuir probe system in two GEC cells is reviewed. The major problems associated with probe diagnostics in a GEC cell are outlined and discussed. While the data base is still insufficient to give definitive values for many parameters, a number of standard measurements are put forward. The plasma density in argon is 9×10^9^ cm^−3^ (±20 %) at an applied rf voltage of 250 V (500 V peak to peak) and a gas pressure of 13.3 Pa (100 mTorr). The electron density scales linearly with applied voltage. The plasma to ground sheath resistance is shown to be very important with a value of 810 Ω in argon at a pressure of 13.3 Pa (100 mTorr) and discharge current of 0.1 A. The value of plasma to ground resistance scales inversely with discharge current and sublinear with pressure. Two standard features in the electron energy distribution function (EEDF) have been proposed as a test of the ability of a probe system to resolve features, first, the transition from a low temperature (<1 eV) bi-Maxwellian distribution to a Druyveysten distribution (3 eV) at 13.3 Pa (100 mTorr) in argon, and the “hole” in the EEDF at 2 eV to 4 eV in nitrogen plasmas.

## 1. Introduction

This paper provides a description of Langmuir probe studies on the Gaseous Electronics Conference (GEC) reference cell. The GEC cell [1] is designed to provide an experimental platform for comparing plasma measurements carried out in a common reactor geometry by different experimental groups, thereby enhancing the transfer of knowledge and insight gained in rf discharge studies. The discipline applied to the study of GEC cells has had an impact on the quality of work in non GEC reactors and led, in general, to an increase in the accuracy of comparisons and understanding of parallel plate reactors. This paper briefly examines the extension of Langmuir probes to the study of planar inductively coupled plasmas, which will form the next generation of GEC cell studies.

Langmuir probes, in principle, provide a simple and relatively cheap diagnostic for measuring the plasma parameters in low-pressure discharges such as those of interest to the GEC community. However, there are a number of issues in the design and interpretation of Langmuir probe characteristics which have led in the past to a wide disparity in measured parameters obtained under similar conditions. Part of this difficulty results from an imprecise knowledge of the rf discharge parameters, voltage, current and deposited power, but this has been partially resolved by the methodology developed by the GEC community to make accurate measurements of the discharge parameters.

Modern Langmuir probe systems are quite complex diagnostic systems with many features to prevent the pitfalls of probe diagnostics; some of which are less than obvious to the non-expert. Even with these precautions the probe system will be limited to a range of plasma conditions where accurate results are obtainable. A probe system in a GEC cell can provide the following parameters: floating potential, *V*_f_, plasma potential, *V*_p_, electron density, *N*_e_, ion density, *N*_i_, electron temperature, *kT*_e_, and electron energy distribution function (EEDF). The probe can obtain data as a function of position with a resolution of a few mm and in time with temporal resolution of few µs. The order of presentation of the parameters above can loosely be regarded as in order of difficulty, with the floating potential being the easiest to obtain and the EEDF being the most complex. This paper proposes a number of benchmark EEDF, the two-temperature structure in the case of the low-pressure rf discharge in Ramsauer-type gas such as argon [2,3] and the EEDF of a molecular gas which has a characteristic “hole” in gases such as nitrogen [4] due to the large in-elastic cross-section associated with vibrational excitation. These features are well characterised but difficult for a probe system to resolve and can therefore act as bench-marks on the “quality” of a probe system. There are very few rf Langmuir probe systems discussed in the literature which can in fact resolve such delicate structure in the EEDF.

Careful design of the rf discharge experiment can go some way to alleviate some of the problems associated with a Langmuir probe system [5], but this is of little use to the GEC cell user who must operate the probe system in an existing chamber. Therefore, we will present here details of the rf probe system which has been used over the past decade at Dublin City University (DCU) and has recently been successfully applied to a number of GEC cells. The objective of any probe system design is that the system can be operated by an inexperienced operator and give reliable results: This goal has not yet been fully realised, but the system described here goes a long way toward that aim.

In a truly symmetrical discharge, both the electrodes are the same area and a balanced or symmetrical voltage with reference to ground is applied to the electrodes. While it is possible to construct such a system, the GEC cell is generally operated in both an asymmetrical electrical and asymmetrical geometric configuration. The area of the grounded electrode often includes the chamber walls and faraday shields and thus the grounded electrode greatly exceeds the area of the driven electrode. The applied rf voltage is not balanced and the driven electrode is biased mostly negative with respect to ground.

The driven electrode adopts a DC bias [6]. This is due to the fact that ions flow unimpeded by the time-varying electric fields from the plasma to both electrodes. Thus, as Ref. [6] shows, the plasma potential is related to the DC bias: When the ratio of driven electrode area, *A*_c_, to ground electrode area, *A*_a_, is very small the DC bias approaches, *V*_0_, the amplitude of the driving rf voltage and the average plasma potential approaches zero. When the discharge is symmetrical *A*_c_/*A*_a_=1 the DC bias approaches zero and the average plasma potential approaches *V*_0_/2. While Ref. [6] does not derive the amplitude of fluctuation of the *V*_p_, it is reasonable to argue that the amplitude of fluctuation in *V*_p_ relative to ground will be large when *A*_c_/*A*_a_=1, and will become smaller as *A*_c_/*A*_a_ approaches zero. This follows from the fact that *V*_p_ normally remains positive with respect to ground, but approaches ground at some point in the rf cycle.

## 2. Description of the Langmuir Probe System

### 2.1 Passive Probe Compensation

The asymmetry in the GEC cell is important as it helps to reduce one of the major sources of error in the measurement of Langmuir probe characteristics in an rf plasma, the rf fluctuations of *V*_p_ relative to ground. The removal of rf fluctuation in *V*_p_ relative to ground is normally achieved by means of the Passive Probe method first developed by Gagne and Cantin [7]. The probe is “forced” to float at the rf potential by ensuring that the probe-plasma impedance, *Z*_p_, is much less than the probe-ground impedance, *Z*_s_. The probe circuit is then a potential divider with the rf potential appearing across *Z*_s_ and the probe floats relative to the plasma potential. In the DCU probe we use a series of miniature self-resonant coils to achieve impedance, *Z*_s_>100 kΩ at 13.56 MHz and *Z*_s_>10 kΩ at the 2nd and 3rd Harmonics. The probe-plasma impedance, *Z*_p_, is calculated to be in the region of a few hundred ohms at 13.56 MHz: This is achieved by making the probe holder close to the probe tip of conducting material and capacitively coupling it to the probe. This shield has an area of approximately 2 cm^2^ and is made of stainless steel. This shunt capacitor, C_2_, dominates the plasma-probe impedance but has no effect on the direct current collected to the probe, it is doubtful that the conducting wall of the probe holder will deplete the plasma to a much greater extent than an insulating wall as neither draw a direct current. Figure 1a shows a simple schematic of the DCU probe and Fig. 1b an equivalent circuit of the probe. Note that there is a parasitic capacitance *C*_s_ between the probe surface and the ground. This is normally shielded by the plasma and ignored by most authors. However, if the tuned inductors are located outside the plasma then *C*_s_ is no longer negligible. In the case of glass walled chambers it is conceivable that the blocking inductors can be placed outside the plasma region, this is not possible in a metal walled chamber. A value of *C*_s_ of 0.1 pF (*Z*_s_=117 kΩ) will be sufficient to shunt *Z*_p_ and load the probe. For this reason the tuned inductors must always be inside the plasma and as close to the probe tip as possible. Again, we are facilitated by the construction of the GEC cell where plasma extends out from the central region and the wall is a considerable distance from the plasma region.

### 2.2 Probe Cleaning

The probe surface is made of refractory metal, this is so that the probe can be cleaned by heating to white hot by electron bombardment. This is achieved by biasing the probe to + 100 V, and drawing a current of approximately 60 mA, normally in argon this is achieved at pressures of 13.3 Pa to 26.6 Pa (100 mTorr to 200 mTorr) with plasma powers of a few watts. The probe must then be maintained in a clean condition by biasing at a negative potential so that the probe is under ion bombardment. This cleaning approach is adequate for gases such as Ar or H_2_, for more complex chemistries such as found in commercial etch processes a more stringent routine must be applied. Hysteresis in the probe current-voltage characteristic, apparent drifts in the plasma parameters, and excessively high electron temperatures are all indicative of probe contamination problems. The probe will become contaminated if left biased close to plasma potential for more than a few seconds even in Ar and He. For this reason some authors recommend that the probe is scanned in a period of a fraction of a second. While the DCU probe system will allow this, another approach is to scan the voltage in a non-sequential manner, the collection of a full characteristic can take longer than a second but the probe potential is scanned so as to avoid problems due to contamination.

### 2.3 Probe Geometry

The diameter of the probe tip is 0.38 mm, and the probe holder 1.5 mm increasing to 7 mm at the inductors, efforts are currently being made to reduce it to <5 mm. The probe length is typically 10 mm and is designed to prevent any increase in probe collection area by a sputtered conductive layer: This problem often goes undetected as the conductive layers can have high resistance and are “burned-off” during electron collection, however the layer can contribute to ion current collection. The conductive layer appears on the insulator and when a connection is made to the probe tip, the collection area of the probe increases. The connection is prevented by having a recessed gap between probe tip and insulator. The diameter of the probe is chosen as 0.38 mm so that the probe normally operates in a “thin sheath” mode. Orbital limited probe collection regime (OML) has been found to be unreliable in these plasmas: This problem is discussed below. The probe size can not be excessive as it will deplete the plasma. This is not a major problem in the GEC cell with the present probe, as has been confirmed by microwave interferometry, in general as the increasing probe radius approaches the mean free path of the collected species the current to the probe is decreased. The probe can also significantly alter the charge balance in the plasma greatly perturbing the plasma, two probes are the best way to check for such effects as is discussed below: one probe monitors the plasma floating potential while the second probe is introduced and biased to obtain the current-voltage (IV) characteristic. The probe radius just greater than the Debye length (say by a factor of two) is a good compromise.

### 2.4 Reference Probe Compensation for Fluctuations in Plasma Parameters

The presence of noise in the plasma due to power supplies at line frequency, and due to instabilities in the plasma, or just random fluctuations are a major source of error, particularly in the measurement of the EEDF. While smoothing techniques can remove the noise, the fluctuations are convoluted with the characteristic and limit the resolution of the probe system. The DCU probe electronics reduce noise at frequencies below 100 kHz by high speed synchronous measurement of *V* and *I*, a facility to make high speed synchronous measurement of the floating potential of a reference electrode or probe is also included. This second reference technique, see for example Chen, Ref. [8], allows the single probe system to have the same noise immunity as a double probe. It should be noted that parasitic capacitance between the probe power supply and ground will limit the usefulness of an uncompensated double probe at high frequencies and the double probe will not compensate for oscillations at 13.56 MHz as some authors have mistakenly believed. However, the double probe concept which employs a floating probe in addition to the usual biased probe is very effective at removing the effects of low and medium frequency noise in the plasma (*f*<100 kHz) [8]. Further precautions in the electronic design are to optically isolate the measurements systems and have a single earth point at the chamber.

### 2.5 Plasma-Ground Sheath Resistance, *R*_sh_

**Fig. 2:**
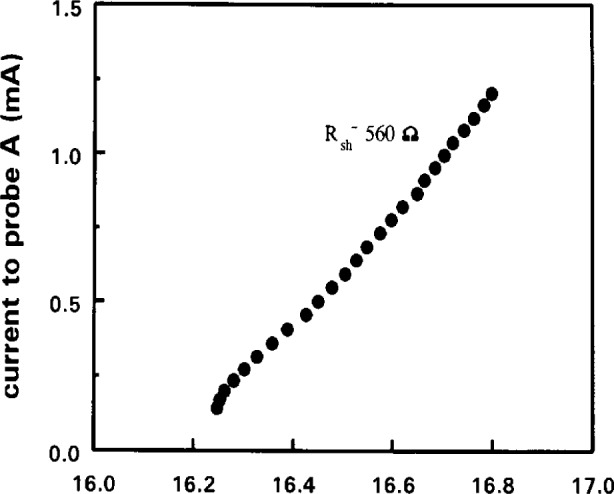
Current to probe versus plasma floating potential (measured by tuned reference probe, *R*_sh_~500Ω).

Finally, one must consider the plasma-ground resistance of the GEC plasma. It is not generally realised that the real resistance between the plasma and the grounded electrode is not negligible and that this resistance is part of the probe circuit. In rf plasmas the plasma-ground impedance is mainly capacitive at 13.56 MHz, the plasma potential is on average many tens of volts above ground potential, and no direct current flows through the discharge. The plasma potential is set by a balance between the ion current to both electrodes [6]. The probe, when drawing electron current, upsets this balance and alters the plasma potential, the change in *V*_p_ as a function of current drawn by the probe is a reasonably linear function and we represent this complex sheath effect by considering the plasma-ground sheath to have an equivalent resistance, *R*_sh_. We can measure *R*_sh_ by using the double probe method described above [8]. The single probe, probe A, is biased and the current voltage characteristic is recorded at the same time the floating potential of probe B is recorded. If we plot the current to probe A versus the floating potential of probe B we obtain a straight line with a slope equal to 1/*R*_sh_, Fig. 2 shows an example of such a plot. Values of *R*_sh_ equal to several kΩ have been measured in the GEC cell, thus a current of a few mA in the probe circuit will cause a shift of several volts in the average plasma potential. This leads to significant errors in many plasma parameters and dramatically alters the shape of the EEDF particularly at low energies. We overcome this problem by, again, using the double probe method; either to measure *R*_sh_ and correct the *I*–*V* characteristic or by using the active synchronous double probe technique to compensate automatically for shifts in plasma potential. We are currently planning to characterise *R*_sh_ for a standard GEC reactor, this will appear in a future publication.

## 3. Langmuir Probe Theory

### 3.1 Laframboise Theory

The analysis routine which is fully automated and is based on the Laframboise [9] theory is described in Ref. [10]. There has been some considerable debate about the suitability of Laframboise theory for probe analysis in the GEC cell. The major drawback in the theory is the requirement that the plasma be free from collisions. This approximation is approached at low pressures but for most of the work in the GEC cell the condition does not hold. Collisions will alter the current collected by the probe. In the ideal conditions, absence of collisions, Laframboise theory is exact, ions are collected on trajectories which intersect the probe, many trajectories miss the probe as the incoming ion has an angular momentum component which originates many tens of Debye lengths from the probe surface. We also assume, *kT*_i_, the ion temperature is zero. Clearly, even in low pressure gases ions will not travel many Debye lengths and conserve their angular momentum. For this reason some authors argue that the simpler theories of radial inward free falling ions to the probe more accurately reflect the real situation [11]. The destruction of the positive ions orbital motion leads to an increase in the collected ion current of a factor of 60 % in the thin sheath situation. In the case of orbital limited collection the destruction of the orbital motion will lead to much more dramatic increases in ion current. Thus, in the thin sheath approximation *R*_p_/*λ*d>1, the effect of weak collisionality is that Laframboise theory overestimates the ion density by a factor of up to 60 %. In general our earlier results [10] in low pressure plasmas tend to show that the measured ion density exceeds the measured electron density by a factor of about 1.6 in argon, even at pressures of 1 Pa (~8 mTorr); we conclude that generally orbital motion is destroyed by collisions but we continue to use Laframboise theory which gives the upper bound on the ion density in the low pressure regime. But, we also note that the ion density is also over-estimated due to the effects of secondary electrons released by photons, metastables and ions striking the probe surface. For this reason the measurement of both electron density and ion density even in an argon plasma is desirable. The weak collisionality discussed here has much less effect on the collection of electrons which are the hotter and lighter species and the electron current close to plasma potential can be measured. This means that sheath expansion contributes much less to current enhancement and therefore the electron density derived here by Laframboise theory is considered much more accurate than ion density, where large sheath expansion has to be compensated by imprecise theory.

It is not possible to avoid the use of complex theories by employing plane probes. The sheath width, *d*_s_, around a probe is of the order of the Debye length, which is 0.074 mm for a typical plasma in the GEC cell, *kT*_e_=1 eV and *n*_e_ =10^10^ cm^−3^ The sheath expands when a voltage is applied, typically the sheath width is given by

$$d_{s}≃\lambda_{d}\sqrt{\left|\frac{V-V_{p}}{kT_{e}}\right|}$$

where *v* is the probe voltage.

Therefore, with a probe biased at −25 V relative to plasma potential, the sheath width will be 0.3 mm. A plane probe will require a radius which is very much greater than the sheath width to allow the application of plane probe theory. Secondly, the potential surrounding a plane probe will extend further into a plasma when plane probes are used and the effects of collisions are more severe.

### 3.2 Effect of Finite Mean Free Path on Probe Theory

**Fig. 3:**
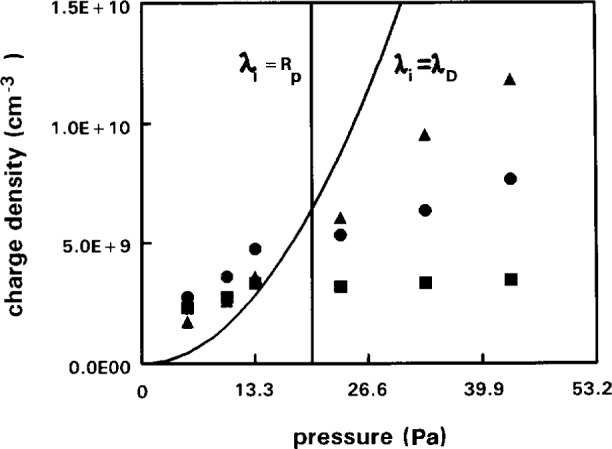
The electron concentration as a function of pressure measured using Langmuir Probe and microwave interferometer in a 200 V argon discharge. ▲—interferometer ●—corrected ion density ■—ion density

At pressures above 6.67 Pa (50 mTorr) the mean free path of ions, *λ*_i_, begins to approach the Debye length and the collection of ions and indeed electrons become influenced by collisions. Of more importance is the fact that the mean free path approaches the radius of the probe, *R*_p_. The presence of the probe alters the density distribution of charged particles, the current to the probe is reduced and the collisionless theory underestimates the charge density. For the probe dimensions given here the probe ion density measurements are accurate to within a factor of 2 up to pressures of about 33 Pa (250 mTorr) in argon, with the density tending to be over-estimated at low pressures and under-estimated at the higher end of the range. Above 33 Pa (250 mTorr) in argon, simple theory [12] estimates that the measured ion density will underestimate the plasma density by a factor which is proportional to *λ*_i_/*R*p, this is confirmed by experiment [14]. Figure 3 shows the plasma density measured by microwave interferometry (absolute uncertainty ±20 % and the ion density measured by the DCU Langmuir probe in a GEC cell. The plot also shows the probe ion density corrected for collisions using the procedure described by Schott [12]. A number of collisional theories exist, see for example the theory of Zakrzewski and Kopiczynski [13], which discuss the issues raised here. The application of a more accurate collisional theory in the interpretation of Langmuir probe results in plasmas in the GEC cell should lead to a dramatic improvement in the usefulness of the probe diagnostic. A more detailed comparison between probe density measurements and Microwave interferometry measurements in the GEC cell is found in a paper by Overzet and Hopkins [14].

### 3.3 Measurement of Electron Density

The measurement of *N*_e_ is in principal less difficult than the ion density as the electron current is measured close to plasma potential and the exact nature of the sheath expansion is not required. The electron current is measured a few volts above the plasma potential and extrapolated back to the plasma potential. This requires an accurate measure of the plasma potential. The electron current drawn is much greater than the ion current so that the possibility that the probe will disturb the plasma is much greater. In general, it is our opinion that the electron density measured by our probe is a lower limit of the plasma density. This is due to the many factors, depletion, electron reflection which all tend to reduce the electron current to the probe. The electron density can also be determined by integrating the EEDF. This method is, in principle, the most accurate method; but close to the plasma potential re-emission of electrons reduce the value of measured *n*(*ϵ*) for energies close to zero energy, where *n*(*ϵ*) is the electron density per unit energy. Above plasma potential re-emitted electrons will be collected. This problem can in part be overcome by extrapolating *n*(*ϵ*) from energies *ϵ*<*kT*_e_/*e* to zero; this procedure is limited by the need for an accurate determination of the plasma potential. We use the intersecting slope method [10], which tends to overestimate the plasma potential.

### 3.4 Influence of RF on Probe Floating Potential

The measurement of probe floating potential is made complex by the presence of a large rf component in *V*_p_. A probe which is allowed to float will actually experience a large amplitude rf voltage applied between the plasma and the ground. To overcome this it is necessary that even a floating probe be tuned as described in Fig. 1. In the absence of a blocking tuned circuit the probe floats close to ground potential, as the impedance to ground is increased the probe floats at a higher potential, typically 10 V to 20 V in the GEC cell. In fact, this is a simple method to compare probes, the probe which floats with the highest floating potential in a similar plasma is the one which is least loading the plasma and therefore has the highest blocking impedance. Ideally the floating potential should be measured by biasing the probe until zero current is drawn, this presents a large impedance to the probe and prevents problems with loading, however, with tuned probes and a high impedance voltmeter very good measurements of the floating potential can be obtained. In the DCU probe system the floating potential is measured by actively biasing the probe to collect zero current (<100 pA): In the double probe configuration the floating probe is connected to a high impedance amplifier, with input impedance >> 10 MΩ, this provides a load on the probe which must draw a few microamps to maintain a high speed synchronous measurement. The extent of loading, which is quite acceptable, can be assessed by comparing *V*_f_ measured by actively biasing the probe and *V*_f_ found by allowing the probe to float connected to the measuring circuit.

## 4. Experimental Results

We will present results from two GEC reactors using the DCU probe system operated by different groups; 1) The first reactor is based at University of Texas at Dallas, (UTD), and is operated by Larry Overzet’s group, the second is the GEC cell at Queen’s Belfast (QUB) operated by the group of Bill Graham. Results from a third reactor, which is a modified etching reactor which is operated by the authors group at Dublin City University (DCU), are not presented in detail but agree well with the GEC results. Three identical probe electronic systems were constructed and operated at the three laboratories. Probes were constructed at each location to the same formula, but slight discrepancies may have occurred.

### 4.1 Floating and Plasma Potential

The floating potential in the GEC cell over a wide range of pressures [6.67 Pa to 66.65 Pa (50 mTorr to 500 mTorr)] and rf amplitudes (100 V to 300 V) is 15 V±5 V in argon, tending to be a few volts higher at higher pressures. This is a good test of the filtering of the probe circuit. If the probe tip is properly decoupled it will float at this value. Distortion due to the presence of rf at the probe-plasma sheath will tend to lower the floating potential of a poorly filtered Langmuir probe. The plasma potential, *V*_p_, is typically ~12 V higher at 28 V±5 V.

### 4.2 Electron Density, *N*_e_

The electron density measured by Langmuir probe (LP) and microwave interferometry (MWI) for argon at 13.33 Pa (100 mTorr) as a function of applied voltage is shown in Ref. [14]. There is good agreement between both techniques which give the electron density in the center of the discharge as 9×10^9^±20 % at 250 V rf amplitude in the UTD GEC Cell. For calibration purposes it is worth noting that *N*_e_ scales linearly with applied voltage up to the highest voltage investigated (300 V). In the QUB GEC cell [16], *N*_e_ was measured at 13.33 Pa (100 mTorr) argon and rf voltage of 138 V is 3.6×10^9^ and at 282 V is 1.3×10^10^ which just agrees within the error, the scaling law established in the UTD GEC cell. The results on the QUB GEC cell are preliminary and we need a larger data base to be more confident that there is good agreement. Further studies [15] show that the electron density increases in the radial direction from the glow center to the electrode radial edge. In this study [15] it is proposed that the electric field is enhanced by the close proximity of the ground shield and the driven electrode at the radial edge. Thus, the current density across the discharge is non-uniform.

At 33.33 Pa (250 mTorr) there is a discrepancy by a factor of almost 2 between MWI and LP, this factor increases to 4 at 66.65 Pa (500 mTorr), consistent with the *λ*e/*r*_p_ scaling law described earlier. In nitrogen, where the mean free path for electron/ion-neutral collisions is shorter, the MWI density was a factor of two greater than the LP measurement at 13.33 Pa (100 mTorr). Thus in complex gases, with high electron/ion-neutral collision rates. Langmuir probe measurements of charge density must be carefully analysed with suitable corrections for collisions even in electropositive gases.

### 4.3 Ion Density

In argon the ion density measurement using Laframboise should agree with the electron density. In general, results in the GEC cell at UDT show that in argon the ion density measurements were 50 % higher than the electron density when *R*_p_/*L*_d_>1 (thin sheath) at low pressures. At higher pressures when the current to the probe was completely collision dominated (>>13.33 Pa (100 mTorr)) the ion density measurement using Laframboise theory agreed remarkably well (within ±20 %) with the electron density in both argon and nitrogen. It is not clear why this should be so, assuming the MWI measurements of *N*_e_ to be accurate it appears that LP measurements of *N*_e_ and *N*_+_ both scale with *λ*e/*R*_p_.

### 4.4 EEDF Measurements in GEC Cells

**Fig. 4:**
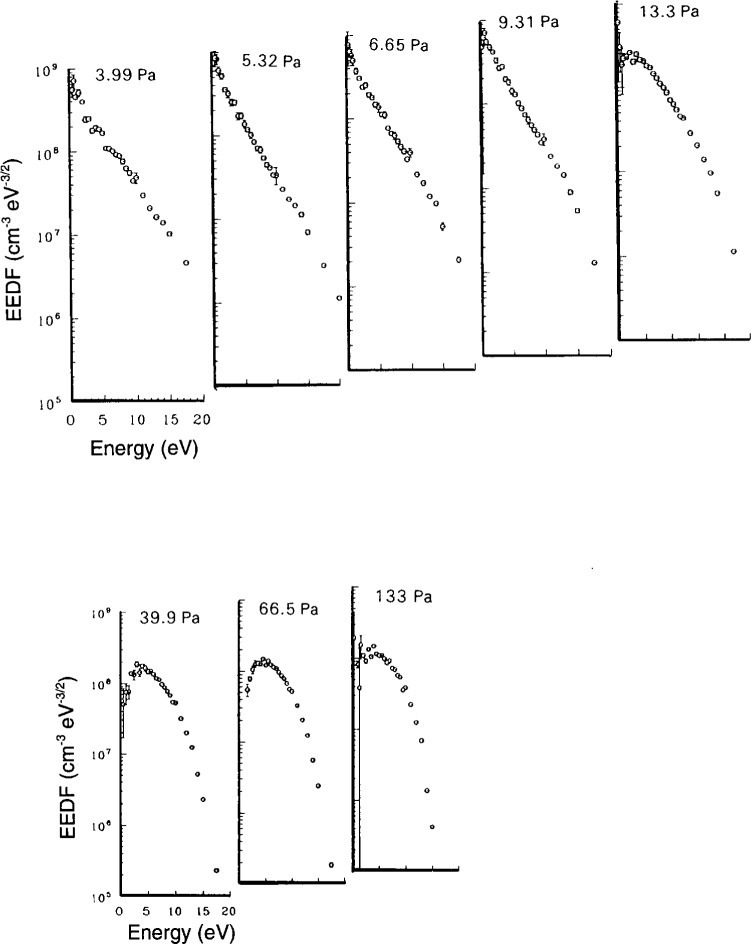
EEDFs in argon in the UTD GEC reference cell. All the data is taken for a current of 175 mA and electrode gap of 25.4 mm.

**Fig. 5:**
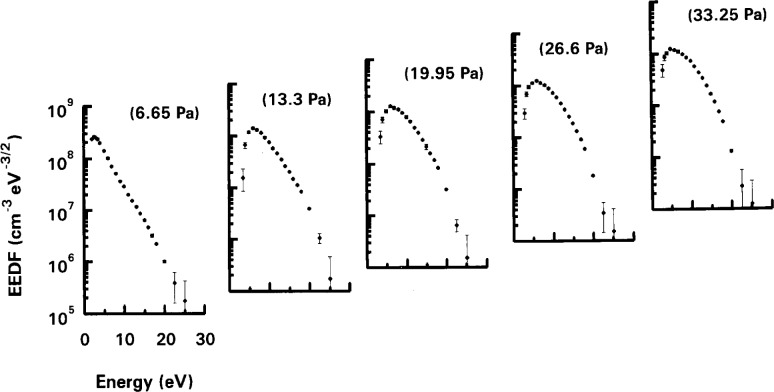
EEDFs in argon in the QUB GEC cell at different pressures and constant power of 10 W, the rf filters are inside the electrode area, no correction for *R*_sh_ is used.

**Fig. 6:**
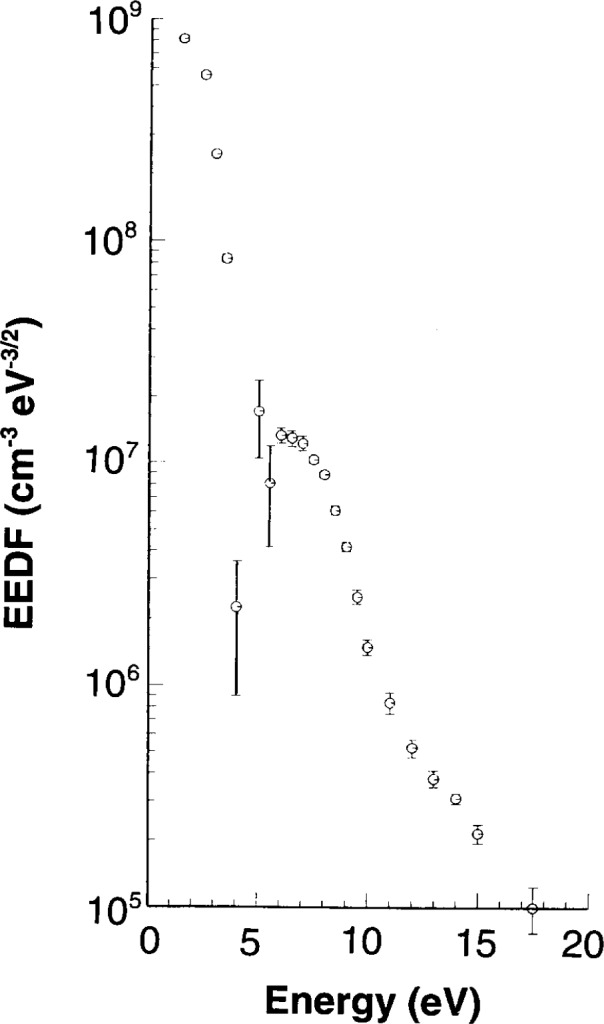
Nitrogen EEDF at 26.7 Pa (200 mTorr) and 175 mA in UTD GEC.

The ultimate test of a probe system is the ability to measure accurately the EEDF. This is the most difficult measurement, but it has been established that there are two calibration checks that can be used to gauge the quality of a probe system. The first is the bi-Maxwellian to Druyveysten transition which occurs in Ar [2] at about 13.33 Pa (100 mTorr) for the GEC cell. Figure 4 shows this transition measured in the UTD GEC cell using the DCU probe. Figure 5 is a similar measurement taken at constant power (10 W) rather than constant current, but showing the same trend in EEDF. The QUB results are not corrected for *R*_sh_ (see Sec. 4.5) and fail to resolve the low energy group, there is also an apparent shift in energy of the EEDF to the right. The second benchmark feature which can be seen in the EEDF of a molecular gas which has a characteristic “hole” in gases such as nitrogen [4] due to the large in-elastic cross-section associated with vibrational excitation. Figure 6 shows the EEDF taken in the UTD GEC cell in nitrogen at 26.66 Pa (200 mTorr) and 175 mA, which agrees with the results in the DCU etcher.

### 4.5 Measurement of Plasma-Ground Resistance, *R*_sh_

**Fig. 7:**
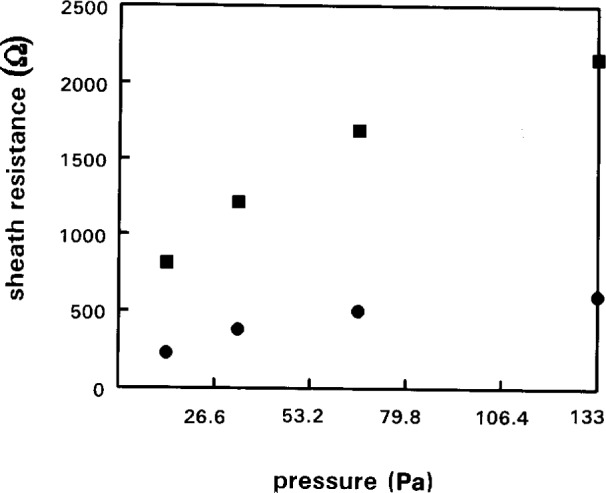
*R*_sh_ measured in the UTD GEC cell using tuned Langmuir Probe with additional reference probe as function of pressure (100 mTorr=13.33 Pa). ■—0.1 A discharge current ●—0.3 A discharge current.

The resistance of the plasma-ground sheath appears in series with the probe circuits. It is therefore an essential parameter to know in order to make reliable measurements of the true *I*–*V* characteristics of the plasma. The DCU probe system operates with a second reference probe to either measure the value of *R*_sh_ or automatically compensate for shift in *V*_p_. Figure 7 shows the *R*_sh_ measured in the UDT GEC cell using the DCU single probe with reference probe. The sheath resistance increases sublinear with pressure and is inversely proportional to discharge current. This scaling appears quite general and applies even to a parallel plate etcher examined at DCU. The value of *R*_sh_ is uniquely defined by the discharge current and pressure for a particular gas.

### 4.6 Spatial Variation of Plasma Parameters

Measurement of the spatial variation of plasma parameters have been made successfully [15]. These show that the plasma radial density peaks near the edge of electrodes, with a local minimum in the centre. The axial variation shows a peak in the centre decreasing as the sheaths are approaching. The difficulties associated with spatial measurements are that the inductors must always be contained with the plasma, to reduce the stray capacitance between the probe tip and ground. In addition, the probe will be greatly perturbed if the shield or tip enter a sheath region.

## 5. Langmuir Probe System in Transformer Coupled Discharges

**Fig. 8:**
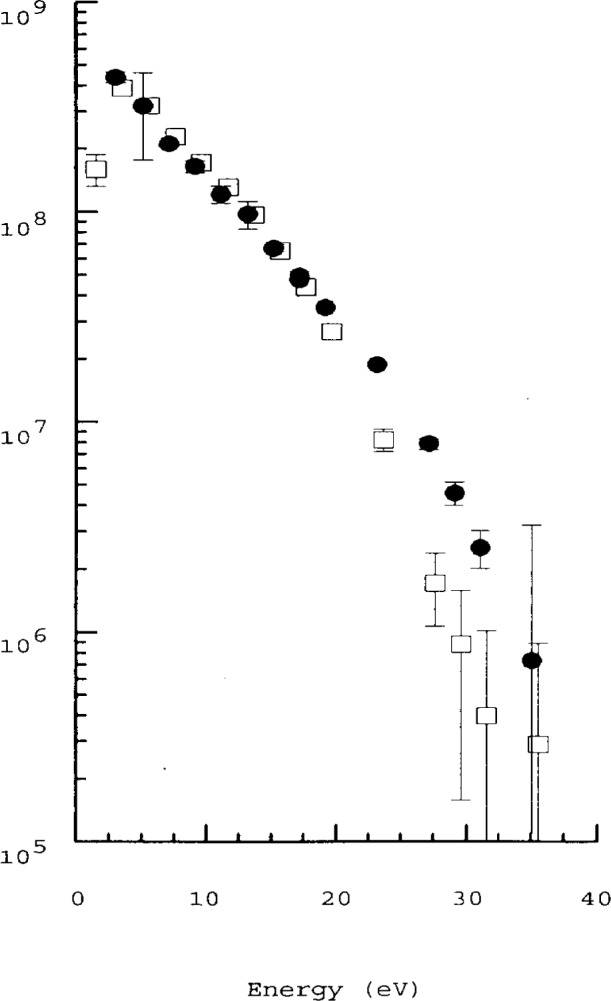
The electron energy distribution function, in a TCP 10 mm from the antenna coil, measured by a plane, single side probe. ●—The probe is oriented so as to intersect the induced current in the plasma. □—The probe is orientated parallel to the induced plasma current.

The next generation of GEC cell will be based on the inductive, or transformer coupled discharge (TCP) where the plasma is sustained by the E-Field generated by a coil placed above the discharge region. The author is not aware of any results of the probe system described here on TCP GEC cells. However, the probe system has been run very successfully in TCP plasmas at DCU. The higher plasma densities and higher electron temperature mean that, in principle, the Langmuir probe measurements are made easier. *R*_sh_ is much smaller and can be ignored for most purposes. The problems which arise in the TCP are that spluttering of material from the window in front of the coil will coat the discharge chamber wall with insulating material and lead to a reduction in the return path for direct current in a single probe system. There are very significant currents flowing in a TCP plasma, the currents are several orders of magnitude higher than in a parallel plate discharge and mean that the EEDF is no longer isotropic. The larger currents to probes means that excessive voltage on the probe can erode the probe surface, or damage the passive circuits used to eliminate rf fluctuations in the plasma potential due to residual capacitive coupling. The use of plane probes is preferred in a TCP. We use a single sided plane probe. Figure 8 shows a typical EEDF taken in the TCP based at DCU. The probe is oriented (i) parallel to the induced current and (ii) perpendicular to the induced current. The single faced probe rectifies the current and clearly displays the anisotropic nature of the EEDF [17]. While the EEDF is no longer isotropic, the term is still used to describe the measured distribution.

## Figures and Tables

**Fig. 1(a):**
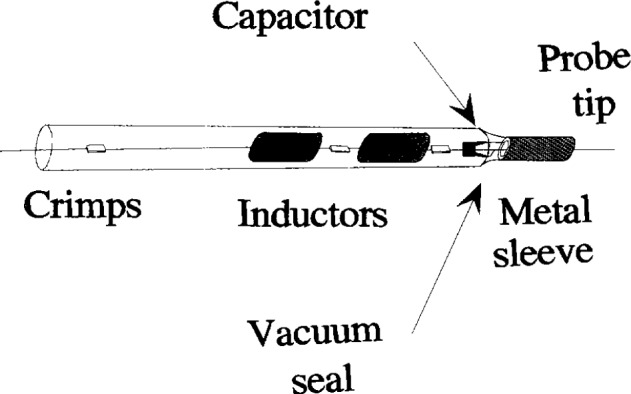
The tuned probe.

**Fig. 1b:**
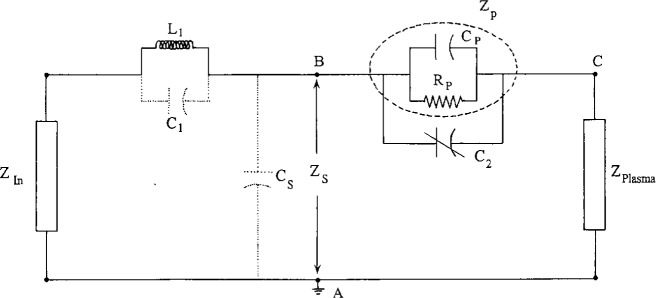
Equivalent circuit of the tuned probe.
